# Epidemiological trends for hospital admissions for acute rotavirus gastroenteritis in Belgium following the introduction of routine rotavirus vaccination and the subsequent switch from lyophilized to liquid formulation of Rotarix^™^

**DOI:** 10.1017/S0950268816001151

**Published:** 2016-07-04

**Authors:** M. RAES, D. STRENS, J. KLEINTJENS, E. BIUNDO, T. MOREL, A. VYSE

**Affiliations:** 1 Jessa Hospital, Pediatrics, Hasselt, Belgium; 2 Realidad, Health Economics, Grimbergen, Belgium; 3 Deloitte, HEOR, Diegem, Belgium; 4 Clinical Pharmacology and Pharmacotherapy, KU Leuven, Belgium; 5 GSK Vaccines, Wavre, Belgium

**Keywords:** Acute, Belgium, epidemiological, gastroenteritis, Rotarix™ vaccination, rotavirus, trend

## Abstract

This study describes epidemiological trends for acute rotavirus gastroenteritis (RVGE) in Belgium in children aged ⩽5 years during the period June 2007 to May 2014 after the introduction of routine rotavirus (RV) vaccination. This period encompassed the switch from lyophilized to the liquid formulation of Rotarix*™* (GlaxoSmithKline, Belgium) in August 2011. Uptake of RV vaccine remained consistently high throughout the study period with Rotarix the brand most often used. RV was present in 9% (1139/12 511) of hospitalized cases with acute gastroenteritis included in the study. Epidemiological trends for hospital admissions for RVGE remained consistent throughout the study period, with no evidence of any change associated with the switch from lyophilized to liquid formulation of Rotarix. This suggests both formulations perform similarly, with the liquid formulation not inferior regarding ability to reduce hospital admissions for acute RVGE in children aged ⩽5 years. A strong seasonal effect was observed with most RVGE occurring in the winter months but with some variability in intensity, with highest incidence found in those aged 6–24 months. The main observation was the decreased number of hospital admissions for RVGE in Belgium that occurred during winter 2013/2014.

## INTRODUCTION

Rotavirus (RV) is an important cause of severe gastroenteritis (GE) in young children aged ⩽5 years, with the epidemiology in Western Europe well described [1, 2]. Two RV vaccines are currently licensed (Rotateq*™*; Merck & Co. Inc., West Point, USA and Rotarix*™*; GlaxoSmithKline, Belgium) and are included in routine childhood immunization schedules [3]. While originally only available in lyophilized form, a liquid formulation of Rotarix was subsequently developed to facilitate handling for vaccine administration and reduce shipment and storage costs. It contains the same virus strain at the same viral concentration and clinical trials show a similar immunogenicity and safety profile to the lyophilized formulation [4, 5]. Both Rotarix (since November 2006) and Rotateq (since June 2007) are currently available as part of the national routine immunization schedule for Belgian infants, and in August 2011 the liquid formulation of Rotarix replaced the lyophilized formulation in Belgium. Currently Belgium does not generate routine national vaccine coverage estimates for those RV vaccines available in its infant immunization schedule. However, Belgium is considered a country that achieves a high uptake (>90% coverage) of RV vaccines in the target age groups [3], with WHO estimates also available for RV immunization coverage in Belgium that support a high uptake [6].

While RV vaccines do not completely prevent the risk of RV infection they are highly effective in preventing RV infection severe enough to require hospitalization [7]. The reduction in acute rotavirus gastroenteritis (RVGE) following the introduction of routine immunization with RV vaccines and the public health impact these vaccines have had on this potentially serious childhood disease is now well established in both Belgium and other European countries, and is described elsewhere [3, 8].

This observational study describes epidemiological trends for acute RVGE in Belgium in children aged ⩽5 years following the introduction of RV vaccines. It covers an extended period from the beginning of June 2007 to the end of May 2014 and includes the switch from lyophilized to liquid formulation of Rotarix in August 2011. This therefore allows epidemiological trends for acute RVGE to be compared before and after this switch for any differences that may suggest that the ability to protect against acute RVGE differs between the two formulations. In the absence of routine national vaccine coverage data, this study also uses Intercontinental Marketing Services (IMS) health sales data to provide an insight into both the uptake of RV vaccines across Belgium during the study period and the extent to which each of the two available brands (Rotarix and Rotateq) were used.

## METHODS

This study used an observational, ecological design as recommended by CDC/WHO for monitoring the impact of RV vaccination on GE disease burden [9–11]. Relevant data were obtained from hospital records for children aged ⩽5 years who had the opportunity to be routinely vaccinated with Rotarix (i.e. born on/after 1 September 2006) and were hospitalized with acute GE and provided a stool sample for a RV detection test. Data collected from hospitalized subjects with acute GE included date of birth, gender, date of stool sample, date of hospitalization, date of discharge and result of the laboratory test for RV. No patient-identifying information was used in this study.

Data were collected from eight Belgian hospitals (two university hospitals and six regional hospitals) during the period 1 June 2007 to 31 May. Two were paediatric hospitals specifically and six were general hospitals with a paediatric ward, and together represented seven (Flemish-Brabant, Namur, East-Flanders, West-Flanders, Limburg, Antwerp, Hainaut) of the ten Belgian provinces plus the Brussels-Capital region. All candidate data available from participating hospitals throughout the study period that met the necessary inclusion criteria were periodically retrieved retrospectively in an aggregate format and checked for double entries. Each hospital also reported any changes made during the study period to those laboratory assays routinely used to confirm the presence of RV in stool samples.

Data were analysed descriptively stratifying results by age, gender, participating hospital, Belgian region and calendar time using tables and graphs. Association between the likelihood of a sample testing positive for RV following hospital admission for acute GE and age group (in months), calendar month of admission, participating hospital, gender, and time period (stratifying by when either the lyophilized or the liquid formulation of Rotarix were in use) was investigated by univariate analysis using *χ*^2^ tests. A multivariable analysis was also undertaken using multinomial logistic regression to investigate for any evidence of a change in epidemiological trend for hospital admissions for RVGE following the switch from lyophilized to liquid formulation of Rotarix in August 2011. The laboratory test result for RV following hospital admission for RVGE was used as the dependent variable and the effect of age (continuous), calendar month (categorical), participating hospital (categorical) and time period pre- or post-introduction of the liquid formulation of Rotarix (categorical) were included as exploratory variables. Logistic regression was also used to further investigate any possible change following the introduction of the liquid formulation by additionally including a 12-month transition period as a further explanatory variable following the introduction of the liquid formulation in August 2011. The purpose was to explore the possibility that both formulations of Rotarix may have been available and in use for an extended period while stocks of the lyophilized formulation were used up, potentially affecting any differences in epidemiological trends that may be associated with each of the two formulations. All quantitative analyses were performed using R software v. 0.98.1028 (https://www.rstudio.com/products/rstudio/).

IMS health sales data for Belgium were accessed for the full study period and used to provide insights into vaccine uptake, with results stratified by RV vaccine brand (Rotarix and Rotateq) and calendar time [12].

## RESULTS

During the entire study period, data were obtained for a total of 12 511 children aged ⩽5 years admitted to hospital with acute GE who had stool samples tested for the presence of RV. The majority (68%) were children aged <1 year with males making up approximately half (54%) of the study population. Each participating hospital contributed data from between 145 and 2751 subjects with acute GE during the study period, with seven of the eight hospitals each providing data for >900 subjects meeting the necessary inclusion criteria. Seven of the participating hospitals reported using rapid immunochromatographic tests to confirm a RV infection, with one hospital using an immunofluorescence assay. Changes in the use of laboratory assays used reported during the study period reflected changes in product manufacturers rather than changes in the preferred diagnostic testing method. Therefore the laboratory methodology used throughout the study period was considered consistent for each participating hospital.

**Fig. 1.**
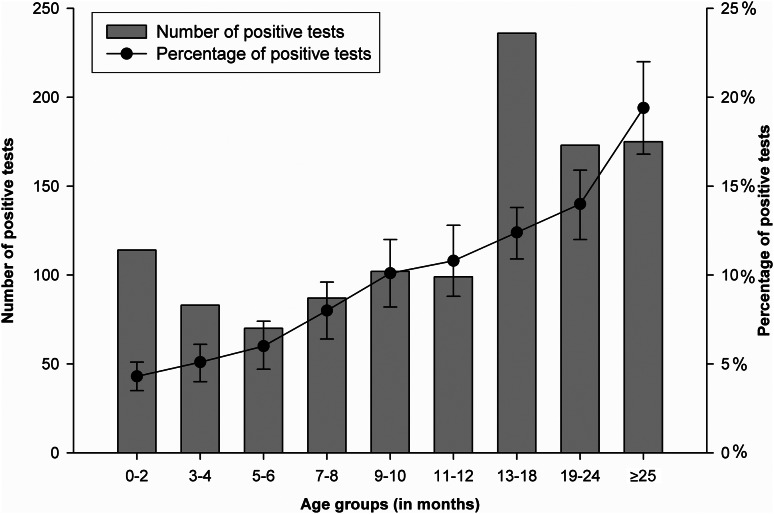
Number of positive tests and proportion (with 95% confidence intervals) testing positive for rotavirus stratified by age group (months).

**Fig. 2.**
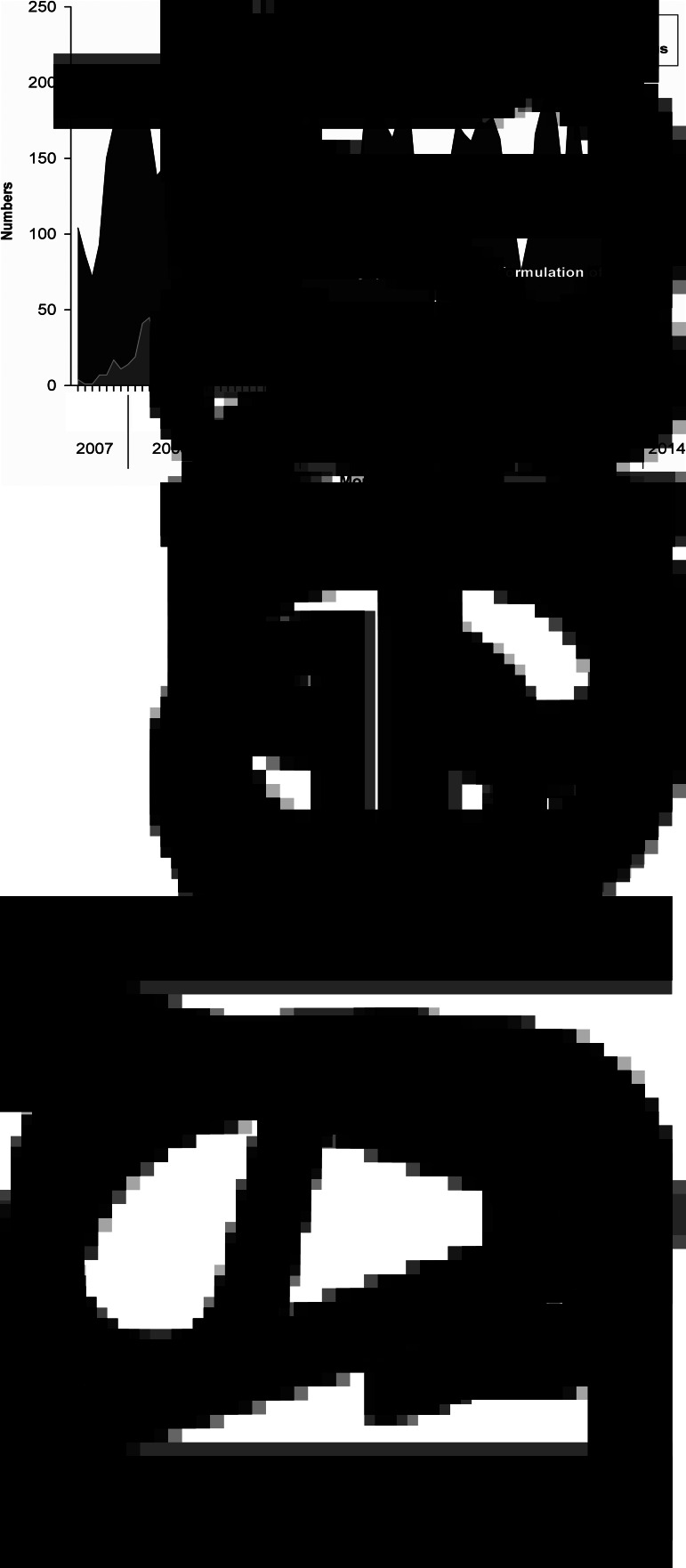
Number of rotavirus laboratory tests performed and number of samples testing positive stratified by calendar month across the entire study period

**Fig. 3.**
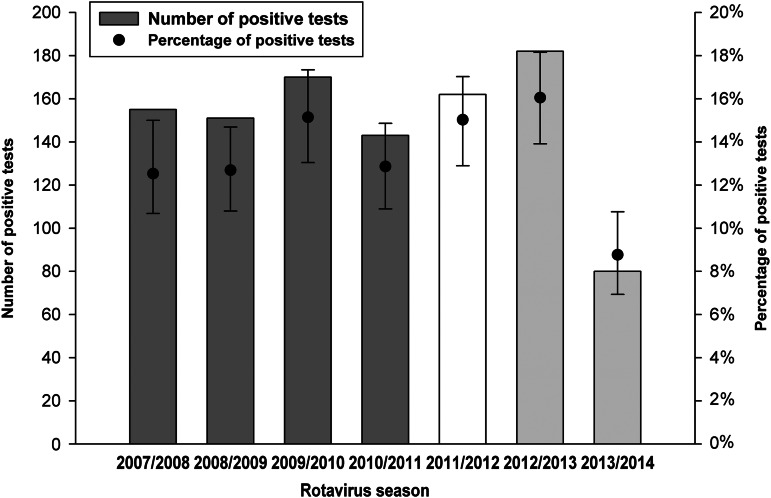
Number and proportion of samples testing positive for rotavirus by individual rotavirus season during the study period (with 95% confidence intervals). The dark and light grey bars reflect use of the lyophilized and liquid formulations of Rotarix, respectively. The white bar reflects the season in which the switch from lyophilized to the liquid formulation occurred.

**Fig. 4.**
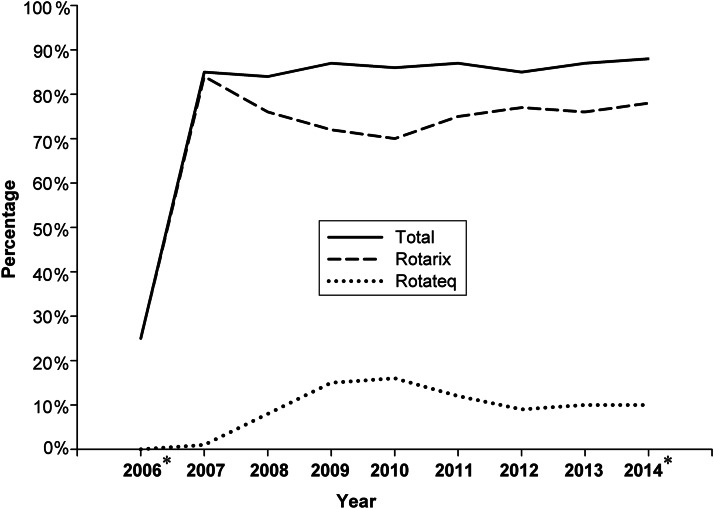
Rotavirus vaccine uptake in Belgium (% of newborns vaccinated) over time stratified by vaccine brand (Intercontinental Marketing Services health sales data). * Denotes those years where data were not used for all the 12 months (reflecting the study period which began in September 2006 and concluded in May 2014).

Overall, the presence of RV was laboratory confirmed in 9% (*n* = 1139) of the study population. The age distribution of samples tested was similar for each participating hospital during the study period. Figure 1 shows how the number and proportion of samples testing positive for RV varied by age group. Table 1 further stratifies these data by period when each formulation of Rotarix was in use, and illustrates that the proportion of samples testing positive by age group was very similar for each formulation. Figures 2 and 3 illustrate seasonal trends and fluctuations for samples testing positive for RV over time. Figure 2 shows both the number of samples tested and the number testing positive for RV by calendar month across the entire study period. Figure 3 presents the number and proportion of samples testing positive by ‘RV year’ across the study period. A ‘RV year’ was defined as a 12-month period from November to October the following year, consisting of a peak season from November to May when the majority of cases occur and a corresponding low season from June to October. Figure 4 shows uptake of RV vaccine in Belgium (stratified by brand) by calendar year using IMS health sales data.

**Table 1.** Number and proportion of samples testing positive for rotavirus stratified by age group and period when each Rotarix formulation was in use

Age group (months)	Lyophilized tests			Liquid tests		
	No. of tests	No. of pos. tests	% pos. tests (95% CI)	No. of tests	No. of pos. tests	% pos. tests (95% CI)
0–2	1738	70	4·0 (3·2–5·1)	621	31	5·0 (3·4–7·0)
3–4	1043	51	4·9 (3·7–6·4)	428	25	5·8 (3·8–8·5)
5–6	733	51	7·0 (5·2–9·0)	281	11	3·9 (2·0–6·9)
7–8	688	63	9·2 (7·1–11·6)	278	16	5·8 (3·3–9·2)
9–10	641	70	10·9 (8·6–13·6)	260	19	7·3 (4·5–11·2)
11–12	557	58	10·4 (8·0–13·6)	255	28	11·0 (7·4–15·5)
13–18	1146	146	12·7 (10·9–14·8)	566	57	10·1 (7·7–12·9)
19–24	699	99	14·2 (11·7–17·0)	384	49	12·8 (9·6–16·5)
⩾25	369	68	18·4 (14·6–22·8)	357	73	20·5 (16·4–25·0)

CI, Confidence interval; pos., positive.

Univariate analysis showed clear evidence of an association between the likelihood of a stool sample testing positive for RV following hospital admission for acute GE and age (*P* < 0·001), calendar month (*P* < 0·001) and participating hospital (*P* < 0·001). There was no evidence of an association with gender (*P* > 0·1) or difference in likelihood of RV detection between the two time periods reflecting use of either lyophilized or liquid Rotarix formulation (*P* > 0·1).

Multivariable logistic regression reflected the univariate analysis and provided strong evidence (*P* < 0·001) that the likelihood of testing positive for RV was associated with increasing age, month of sampling and participating hospital. It also provided weak evidence of a reduced likelihood of a sample testing positive for RV during the 33-month period following introduction of the liquid Rotarix formulation [odds ratio (OR) 0·86, 95% confidence interval (CI) 0·75–0·99, *P* = 0·034]. The regression model used to further investigate any possible change following the introduction of the liquid formulation also showed no evidence for any change in the likelihood of a sample testing positive for RV during the 12-month period following the switch to liquid formulation (*P* > 0·1). However, it provided weak evidence of a reduced likelihood of a sample subsequently testing positive for RV during the 21-month period following a 12-month transition period (OR 0·86, 95% CI 0·73–1·0, *P* = 0·048).

## DISCUSSION

The data used in this study are appropriate for undertaking epidemiological surveillance via the monitoring of trends of hospital admissions for acute GE in those aged ⩽5 years where a RV detection test was performed. Both data collection and the RV detection methods used remained consistent throughout the study period, enabling meaningful comparisons to be made. Nevertheless, it is acknowledged that incidence of RVGE is likely to be underestimated as many patients may not seek medical treatment or be hospitalized [1]. This study is therefore most appropriate for describing broader epidemiological trends and highlighting any key or significant epidemiological changes that may have occurred during the study period.

This study used data from a large number of Belgian children (*n* = 12 511) aged ⩽5 years hospitalized with acute GE across Belgium. The only Belgian provinces not represented were Walloon-Brabant, Liege and Luxembourg, which in 2010 were estimated to contain ~16% of the total Belgian population [13]. While the quantity of data from participating hospitals varied, each contributed significantly to the total used with all but one providing data for at least 900 subjects that met the study inclusion criteria. The data used in this study are therefore considered to approximate the Belgian population of children aged ⩽5 years hospitalized with acute GE.

Following the introduction of Rotarix into the Belgian national immunization schedule, IMS health sales data indicated that uptake of RV vaccines has been consistently high in Belgium with no evidence to suggest this changed during the study period [12]. However, these data should be interpreted with some caution as they relate to number of doses sold rather than a specific measure of vaccine uptake, and may overestimate vaccine coverage. These data also provide some insight into the extent to which the two different RV vaccine brands are used, and show that Rotarix has consistently continued to achieve the large majority of the RV vaccine market share in Belgium since it began to be routinely used in 2006. While routine national RV vaccine coverage data for Belgium are not yet available, RV vaccine coverage was estimated to be 92·2% (95% CI 90·2–93·8) in 2012 in a study conducted in the Flemish region alone [14]. In 2010 the Flemish region was estimated to contain ~58% of the total Belgian population [13] and the RV vaccine coverage estimate reflects the conclusions for RV vaccine uptake drawn from this analysis using IMS health sales data. Moreover, these data are also consistent with estimates of RV immunization coverage for Belgium made by the WHO [15]. Therefore, given the high uptake of RV vaccines in Belgium and the predominance of Rotarix, the epidemiological trends observed for acute RVGE during the study period are most likely to have been influenced by use of Rotarix. RV was not detected in the large majority (91%) of samples from children hospitalized with acute GE that were used in this study. This is consistent with the high uptake of RV vaccine achieved in Belgium which, while not considered to completely prevent the risk of RV infection, is effective in preventing severe RV infection [7].

A marked cyclical seasonal trend was identified, with the majority of laboratory-confirmed RV cases occurring during the winter and spring months. This occurred consistently across the entire study period, although some variability in intensity was apparent with more cases detected during some seasons than others. This temporal effect with the majority of RV cases in Western European countries occurring between November and May is well documented [1, 16]. However, these Belgian data are particularly notable for a substantial reduction in the number of hospitalizations with RVGE observed during the 2013/2014 winter season, with a comparatively high number of samples testing positive for RV during the preceding winter of 2012/2013. An unexpected significant fall in RV detections was also observed during the 2013/2014 winter in The Netherlands, a neighbouring country where routine vaccination against RV is not yet in place [17]. This suggests that rather than being a phenomenon specifically associated with Belgium, an unusual RV epidemiology characterized by a lower than usual incidence may have been affecting parts of Western Europe more widely during the 2013/2014 season. The extent of this phenomenon will be able to be better assessed as further country-specific RV surveillance data in the European region become available for this period. However, it is speculated that the exceptionally mild European winter of 2013/2014 with average daily temperatures significantly higher than normal may have contributed to reduced RV transmission in the region [17, 18]. It has also been suggested that the recent introduction of RV vaccination in both the UK and Germany in 2013 may have contributed to a low incidence in neighbouring European countries in 2013/2014 by reducing the number of introductions of RV from these neighbouring populations [17]. Furthermore, a comparatively high RV incidence was also observed in The Netherlands during the 2012/2013 winter, reflecting a similar observation using these Belgian data where an increased number of samples testing positive for RV also occurred during this period, suggesting that this also is likely to reflect a wider epidemiological feature affecting the western European region at this time. This additionally may have contributed to a reduced incidence in Belgium during the 2013/2014 winter through depletion of susceptibles [17]. The reduction in RVGE incidence at this time therefore most probably reflects a wider epidemiological trend affecting the Western European region, and it is very unlikely that these temporal variations in intensity of the RV season observed in Belgium during the winters of 2012/2013 and 2013/2014 are related to the use of RV vaccine in Belgium or the switch from lyophilized to liquid Rotarix formulation in August 2011.

This varying intensity in seasonality is also likely to have influenced the findings of the multivariable analyses where a reduced likelihood of hospital admission for confirmed RVGE was associated with the period following introduction of the liquid formulation of Rotarix. The particularly low numbers of RV cases occurring in Belgium during the 2013/2014 winter most probably resulted in an overall net reduction in the likelihood of a hospital admission for RVGE indicated by the models for the period following the switch to liquid formulation.

These study data focused on those aged ⩽5 years and showed a strong association between RVGE and age, with samples from those aged 6–24 months most likely to test positive for RV. This trend was consistently observed throughout the study period. This closely reflects the findings of a previous study investigating the age distribution of paediatric RVGE cases in Europe (which included Belgian country-specific data) where the majority of RVGE was detected in children aged 6–24 months [19]. The proportion of total samples for each age group testing positive for RV in this study also remained very similar for each period reflecting the use of lyophilized and liquid formulations of Rotarix, respectively. There was therefore no evidence to suggest that the trend by age described using these data changed during the study period. However, in Belgium a RV detection test is only reimbursed for subjects with suspected RVGE if the subject is aged < 24 months, and testing is therefore less likely to be undertaken with samples from older children. Data from relatively few samples from children aged 2–5 years were therefore available for this study, consistent with the bias that is likely to be present in data of this nature where samples from such older age groups are prone to under-representation. Healthcare is also more likely to be sought for younger infants experiencing their first RV infection where symptoms are more likely to be severe, suggesting this may also have increased the number of samples from younger subjects available for use in this study [19]. Caution should therefore be exercised when using data of this nature to investigate associations with age, and they are most appropriate for identifying the presence of major changes in broader epidemiological trend in relation to age group.

Data obtained for this study showed that both admissions where RVGE was laboratory confirmed and the number of RV detection tests performed varied by participating hospital, suggesting the possibility that some regional variation in the epidemiology of RV may be present in Belgium. However, given that Belgium is geographically a small country that has consistently achieved a high national RV vaccine uptake since the introduction of RV vaccines into the routine schedule in 2006 [3, 14], it is very unlikely that these differences reflect true localized regional variations in the epidemiology of RV in those aged ⩽5 years. The differences observed by participating hospitals therefore possibly reflect discrepancies in the willingness and capacity of each to undertake a confirmatory RV detection test.

This study monitored epidemiological trends in hospital admissions for RVGE in those aged ⩽5 years in Belgium for four RV seasons prior to and for three RV seasons after the switch from lyophilized to liquid Rotarix formulation. Sufficient data were therefore available to model and compare the likelihood of a hospital admission for RVGE before and after the switch to Rotarix liquid formulation using logistic regression. Results indicate that following the switch to Rotarix liquid formulation in Belgium in August 2011, there was no evidence to suggest an increased likelihood of hospital admissions for RVGE. On the contrary, the results suggest a decreased likelihood of hospital admissions for RVGE in those aged ⩽5 years following the switch to the liquid formulation. However, this finding was very likely influenced by the significant fall in RV detections that occurred during the 2013/2014 RV season, resulting in an overall net reduction in the likelihood when considering the period following the switch to the liquid formulation. Based on reports from The Netherlands [16], the comparatively low number of hospital admissions for RVGE in Belgium during the 2013/2014 winter is attributed to a likely wider epidemiological phenomenon affecting RV in Western European countries, and is not considered related to specific RV vaccine use in Belgium. The epidemiological trends shown by these data are therefore considered consistent across the study period when each formulation of Rotarix was in use. These observational data therefore provide no evidence to suggest that liquid formulation of Rotarix may be less effective at reducing hospital admissions for RVGE in those aged ⩽5 years compared to the lyophilized formulation, and support the conclusion that it is not inferior to the lyophilized formulation in this respect.
